# Performance of urinary kidney injury molecule-1, neutrophil gelatinase-associated lipocalin, and *N*-acetyl-β-D-glucosaminidase to predict chronic kidney disease progression and adverse outcomes

**DOI:** 10.1590/1414-431X20176106

**Published:** 2017-03-30

**Authors:** G.R. Lobato, M.R. Lobato, F.S. Thomé, F.V. Veronese

**Affiliations:** 1 Programa de Pós Graduação em Medicina: Ciências Médicas, Universidade Federal do Rio Grande do Sul, Porto Alegre, RS, Brasil; 2 Serviço de Nefrologia, Hospital de Clínicas de Porto Alegre, Porto Alegre, RS, Brasil

**Keywords:** Chronic kidney disease, Hemodialysis, Biomarkers, Kidney injury molecule-1, Neutrophil gelatinase-associated lipocalin, N-acetyl-β-D-glucosaminidase

## Abstract

Urinary biomarkers can predict the progression of chronic kidney disease (CKD). In this study, kidney injury molecule-1 (KIM-1), neutrophil gelatinase-associated lipocalin (NGAL), and *N*-acetyl-β-D-glucosaminidase (NAG) were correlated with the stages of CKD, and the association of these biomarkers with CKD progression and adverse outcomes was determined. A total of 250 patients, including 111 on hemodialysis, were studied. Urinary KIM-1, NGAL, and NAG were measured at baseline. Patients not on dialysis at baseline who progressed to a worse CKD stage were compared with those who did not progress. The association of each biomarker and selected covariates with progression to more advanced stages of CKD, end-stage kidney disease, or death was evaluated by Poisson regression. NGAL was moderately correlated (r^s^=0.467, P<0.001) with the five stages of CKD; KIM-1 and NAG were also correlated, but weakly. Sixty-four patients (46%) progressed to a more advanced stage of CKD. Compared to non-progressors, those patients exhibited a trend to higher levels of KIM-1 (P=0.064) and NGAL (P=0.065). In patients not on dialysis at baseline, NGAL was independently associated with progression of CKD, ESKD, or death (RR=1.022 for 300 ng/mL intervals; CI=1.007-1.037, P=0.004). In patients on dialysis, for each 300-ng/mL increase in urinary NGAL, there was a 1.3% increase in the risk of death (P=0.039). In conclusion, urinary NGAL was associated with adverse renal outcomes and increased risk of death in this cohort. If baseline urinary KIM-1 and NGAL predict progression to worse stages of CKD is something yet to be explored.

## Introduction

Although mortality due to advanced chronic kidney disease (CKD) has decreased in the last few years in the USA (1) and Europe (2), CKD continues to cause significant morbidity and mortality around the world (3). Recent epidemiological and mechanistic studies suggest that acute kidney injury (AKI) and CKD are not distinct entities, but are closely interconnected, as CKD is a risk factor for AKI, AKI is a risk factor for the development of chronic kidney damage, and both acute and chronic renal disease are risk factors for cardiovascular disease (4–6). It has long been recognized that end-stage kidney disease (ESKD) is associated with increased mortality, and that old age, diabetes, and cardiovascular disease are the most important coexisting conditions that predict worse outcomes for patients with ESKD (7).

Traditional blood markers (creatinine) and urinary markers (albuminuria) of kidney injury have been used for decades in clinical studies for diagnostic and prognostic purposes. For prevalent nephropathies such as diabetic kidney disease, they remain as useful clinical markers of renal injury and even of CKD progression. However, considering the variety of causes that lead to kidney damage, these markers seem to be neither sensitive nor specific, mainly in the early disease stages (8). Additionally, these markers do not directly reflect injury to renal cell signaling pathways and internal organization. Unlike cardiac troponin, most of these markers represent functional and late consequences of injury, and do not explain the type and intensity of aggression that is taking place in the renal parenchyma (9). Thus, new biomarkers of renal injury and function are required in clinical practice to permit accurate diagnosis, guide treatment, and predict disease progression (10). However, a single biomarker may not be adequate to capture the spectrum of CKD, given the inherent heterogeneity of renal structure and the diverse settings under which kidney injury occurs (11).

Distinct molecules are being investigated in the search for an ideal biomarker that could provide a window for intervention between cell disruption, renal damage, and clinical manifestations. Markers of inflammation (interleukin-18, matrix metalloproteinase-2), glomerular filtration rate (cystatin C), proximal tubular response [urinary liver-type fatty acid binding protein (L-FABP) kidney injury molecule-1 (KIM-1), neutrophil gelatinase-associated lipocalin (NGAL)], and tubular enzymes [N-acetyl-β-D glucosaminidase (NAG)] in serum and/or urine have all emerged as predictors of AKI before renal function decline can be detected clinically (12–19). These biomarkers have been intensively investigated in the differential diagnosis of strucutural and functional kidney lesions, in light of their potential to influence clinical decision-making and patient management in routine practice.

Notably, KIM-1, NGAL, and NAG are urinary biomarkers that reflect tubular damage and AKI, and are earlier and more sensitive markers than plasma creatinine in acute renal failure (20). Urinary KIM-1 and NGAL are especially interesting as they are expressed by tubular epithelial cells in response to injury, and could thus identify patients at risk for CKD once progressive interstitial fibrosis and tubular atrophy develop (21). More recently, there have been reports of increased urinary levels of KIM-1, NGAL, and NAG in patients with CKD, which could be used to predict disease progression, renal function decline, adverse renal outcomes, and mortality (21–25). The finding of urinary biomarkers to accurately grade the risk for CKD progression could be clinically useful, as the serial measurements of one or a composite of such biomarkers would give the clinician important information to plan renoprotection strategies to retard the development of ESKD.

The aim of this study was to ascertain whether urinary levels of KIM-1, NGAL, and NAG correlate with the five stages of CKD, including patients on dialysis, and to determine the association of these biomarkers with the adverse outcomes of CKD progression, ESKD (dialysis or transplantation), and death.

## Material and Methods

### Selection of participants

Between April 2014 and October 2014, 250 patients were selected among individuals representing the five stages of CKD as defined by estimated glomerular filtration rate (eGFR) calculated with the Chronic Kidney Disease Epidemiology Collaboration (CKD-EPI) equation (creatinine based). This cohort was followed prospectively at Hospital de Clínicas de Porto Alegre (HCPA) and at an external private hemodialysis clinic in Southern Brazil, the two units from where the patients were recruited. Patients with ESKD on hemodialysis, except those with no residual renal function (anuria) and those scheduled for a kidney transplant, were also included. This study was approved by the HCPA Research Ethics Committee and performed in accordance with the 1975 Declaration of Helsinki. All participants provided written informed consent prior to enrollment. The Research Ethics Committee is registered with the Human Research Protection Committee of the Brazilian Ministry of Health under Institutional Review Board No. 00000921.

### Clinical assessments

Demographics and baseline clinical characteristics were assessed. Clinical data included age, gender, ethnicity, etiology of CKD, comorbidities such as hypertension (defined as current antihypertensive therapy), diabetes mellitus (defined as current treatment with oral hypoglycemic agents or insulin), and tobacco use. Data on etiology of CKD, comorbidities and tobacco use were extracted from medical reports. Patients with ongoing immunosuppression for active glomerulonephritis, evidence of acute kidney injury associated with active infection, under use of drugs or other nephrotoxic agents, or with conditions that could influence the evolution of CKD such as surgical interventions for obstructive uropathy were excluded.

CKD stages were defined according to KDIGO guidelines and based on eGFR levels as: stage 1: >90 mL·min^-1^·(1.73 m^2^)^-1^; stage 2: 60–89 mL·min^-1^·(1.73 m^2^)^-1^; stage 3a: 45–59 mL·min^-1^·(1.73 m^2^)^-1^; stage 3b: 30–44 mL·min^-1^·(1.73 m^2^)^-1^; stage 4: 15–29 mL·min^-1^·(1.73 m^2^)^-1^; stage 5nd (not on dialysis): 10–14 mL·min^-1^·(1.73 m^2^)^-1^; stage 5d (on dialysis): <10 mL·min^-1^·(1.73 m^2^)^-1^. CKD was established in the presence of structural or functional renal damage (eGFR <60 mL·min^-1^·(1.73 m^2^)^-1^) lasting for more than 3 months.

Patients were followed from April 2014 to November 2015, for a median of 15 months. The patients were scheduled for planned follow-up visits at periodic intervals, according to CKD stage. The primary outcome measure was progression to a more advanced stage of CKD, and the secondary endpoints were development of ESKD (dialysis or transplantation) and death.

### Biomarkers and laboratory measurements

First morning void spot urine samples were collected into sterile cups for measurement of KIM-1, NGAL, NAG, total protein, albumin, and creatinine. These measurements were performed at the Renal Division Laboratory of Brigham and Women’s Hospital, Harvard Medical School, Boston, MA, USA. Urine was subsequently centrifuged at 400 *g* for 10 min at 20–25°C to remove the cellular components and the supernatant was aliquoted in 1.8 mL Eppendorf tubes and frozen within 2 h of collection at −80°C. At the time of assay, samples were thawed, vortexed, and centrifuged at 21,952 *g* for 5 min at 4°C, and assays performed in duplicate for biomarker measurement.

Urinary KIM-1 and NGAL were measured by a microbead-based sandwich ELISA (R&D Systems, USA), and NAG by an enzyme-substrate-based colorimetry assay (Roche Diagnostics Corporation, USA), according to the manufacturer's instructions. The intra-assay and inter-assay coefficients of variation and assay range were respectively: a) KIM-1: 4.2%, 6.7%; 0.097–40 ng/mL; b) NGAL: 3.7%, 6.5%; 0.49–1,000 ng/mL; c) NAG: <2%; <2%; 0.2–52.9 mU/mL.

Urine albumin was measured by an immunoturbidimetric assay and urine creatinine by a kinetic colorimetric assay in an automated analyzer. Urinary albumin, KIM-1, NGAL, and NAG were always related to urinary creatinine. Serum creatinine was measured by the Jaffe reaction (Modular P, Roche Diagnostic, Germany) at HCPA.

### Statistical analyses

Sample size was calculated using WinPEPI version 11.43 (Brixton Health, United Kingdom) software, based on the study of Fufaa et al. (25). For a 5% significance level and a statistical power of 80%, and a relative risk associated with urinary NGAL for development of ESKD estimated at approximately 1.70 (25th percentile of the second tertile with the first tertile as reference), the minimum sample size was set at 242 patients.

Normally distributed data are reported as means±SD, and asymmetrically distributed variables as medians and interquartile ranges (IQRs). The association between continuous variables was assessed using Spearman correlation coefficients. To compare means between groups, the Student *t*-test or analysis of variance (ANOVA) was used. In case of asymmetry, the Mann-Whitney U and Kruskal-Wallis tests were used, respectively. Progression of CKD stage in patients not on dialysis at study entry was determined by the last measured eGFR, resulting in reclassification to a more advanced stage or maintenance of the baseline CKD stage. We searched for an association between the underlying disease (CKD etiology) and the levels of urinary biomarkers in subjects not on dialysis at study entry.

The association of urinary biomarker tertiles with CKD progression and the ESKD or death outcomes in patients not on dialysis at baseline was examined. Additionally, to evaluate the independent effect of age, gender, ethnicity, smoking, CKD etiology, baseline eGFR, and urinary biomarkers on the adverse outcomes CKD progression, development of ESKD (dialysis/transplantation), or death, a Poisson regression model was used to control for confounding factors. Variables with P<0.20 on bivariate analysis were entered into the multivariate model. Data were analyzed using the SPSS Statistics Version 22 software package (IBM Corp., USA).

## Results

Clinical characteristics of the study patients are reported in Table 1. The subjects were predominantly of non-African descent (82.4%), with a mean age of 59±15.3 years. As for CKD etiology, hypertension (36%), chronic glomerulonephritis (24%), and diabetes (22%) were most prevalent. Overall, 11% of patients were current smokers and 24% were former smokers. Of the 250 patients studied, 44% were on chronic hemodialysis. The distribution of CKD stages at baseline of non-dialyzed patients was: stage 1, 7.2%; stage 2, 4.8%; stage 3a, 4.4%; stage 3b, 12%; stage 4, 26.4%; and stage 5nd, 1.6% (Table 1). Most patients (71.6% of the sample) had stage 4 or 5 CKD. After a median follow-up of 15 months, of the 139 patients not on dialysis at baseline, 83.5% still had a functioning kidney. Forty-six percent had progressed to worse CKD stages. Thirteen percent progressed to ESKD and started chronic hemodialysis, of whom 5 received a kidney transplant; 5 patients died. Of the 111 patients on hemodialysis at baseline, 7% underwent kidney transplantation, and 1 was lost to follow-up due to change of address. Six patients were transferred to peritoneal dialysis. Among the 250 patients, there were 18 deaths (7.2%): 13 (11.7%) in the hemodialysis group and 5 (3.6%) patients not on dialysis. The causes of death were cardiovascular in 6 patients (33.3%) and infection/sepsis in another 6 (33.3%); the remainder died from other causes, such as malignancy.

**Table 1:**
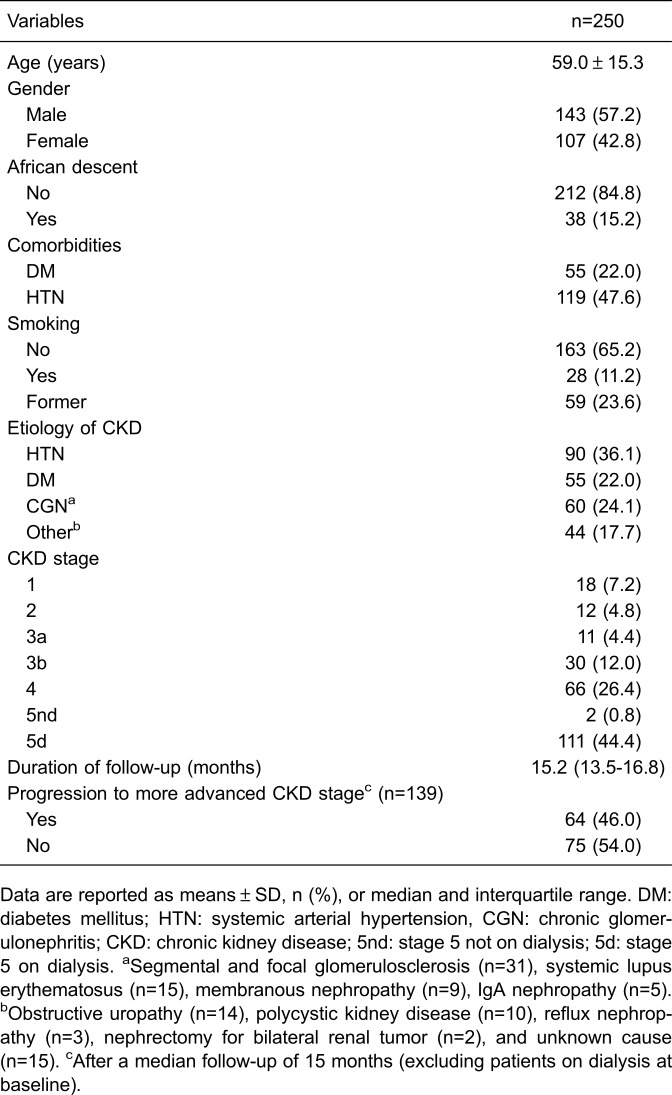
Baseline characteristics of participants with chronic kidney disease stages 1 to 5, including dialysis.

Urinary levels of KIM-1, NGAL, and NAG stratified by CKD stage are reported in Table 2. All biomarkers correlated significantly with CKD stage. The distribution of NGAL correlated moderately with the progressive stages of CKD as determined by the Spearman coefficients. Albeit significant, the correlations of KIM-1 and NAG with CKD stages were weak. The highest levels of the three urinary biomarkers were found in stage 5d.

**Table 2:**
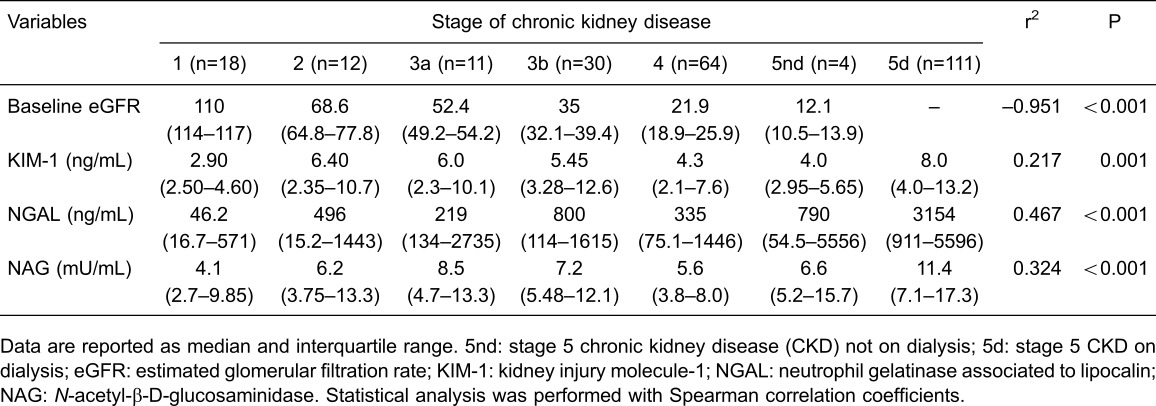
Urinary values of biomarkers according to the stage of chronic kidney disease.

Sixty-four patients (46%) progressed to worse CKD stages (progressors). Baseline eGFR, last eGFR and urinary KIM-1, NGAL, and NAG concentrations of progressors compared to non-progressors are reported in Table 3. Compared to non-progressors, the patients who progressed to worse CKD stages showed a trend toward higher levels of urinary KIM-1 (P=0.064) and NGAL (P=0.065) at baseline; there was no difference in NAG levels.

**Table 3:**
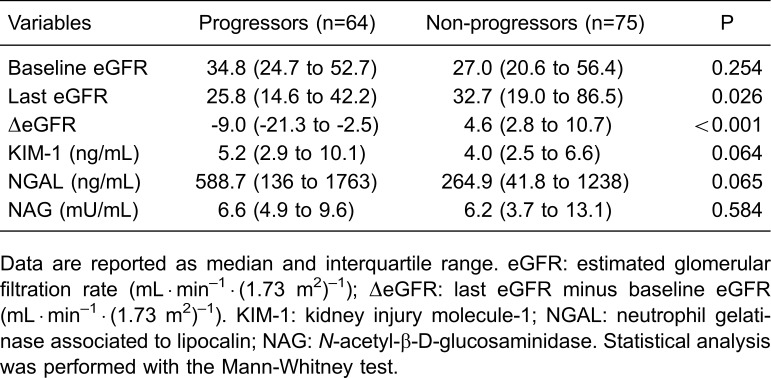
Renal function at baseline and last follow-up and the urinary levels of KIM-1, NGAL, and NAG in patients who progressed to worse CKD stages compared to patients who did not progress.

The association of urinary biomarker tertiles with CKD progression and ESKD or death outcomes in patients not on dialysis at baseline is reported in Table 4. KIM-1 was associated with loss of kidney function and progression to more advanced stages of CKD. Thus, as the rated tertile increased, there was an increased incidence of renal function loss, which was statistically significant for KIM-1 (P=0.047) but not for NGAL (P=0.095). A greater proportion of patients in the highest tertile of urinary KIM-1 levels exhibited progression of CKD (57.8 vs 37% in the lowest tertile, P=0.047) after a median follow-up of 15 months. No association was found between the tertiles of each biomarker and development of ESKD or death.

**Table 4:**
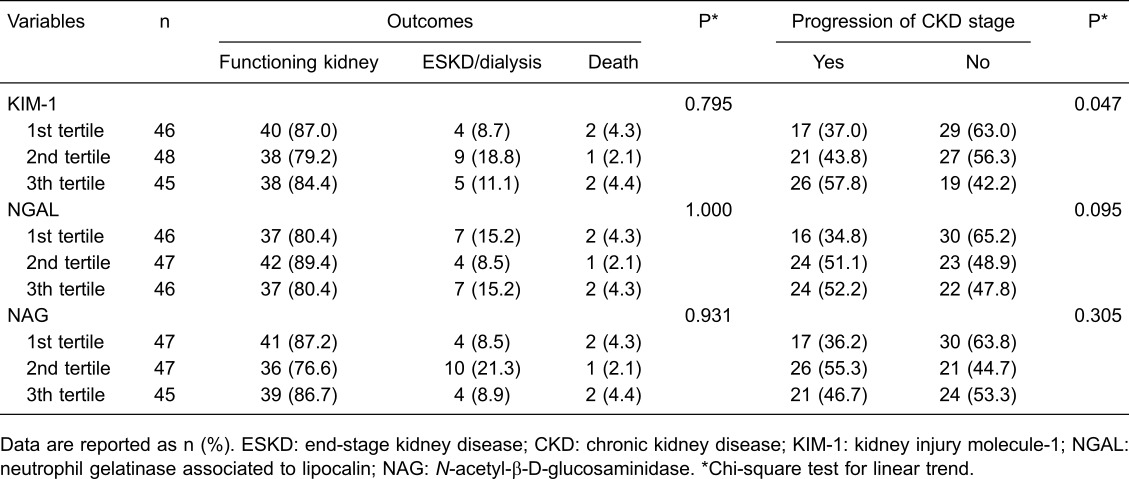
Association between biomarkers (stratified into tertiles) and outcomes in patients not on dialysis at baseline.

We also searched for a potential association between underlying disease and urinary biomarkers in subjects not on dialysis at study entry. Patients with a diagnosis of conditions not related to arterial hypertension, diabetes mellitus, or chronic glomerulonephritis as the etiology of CKD had significantly lower levels of urinary KIM-1 (P=0.011), NGAL (P=0.011), and NAG (P=0.033; Table 5). Comparing urinary levels of each marker across the most prevalent CKD etiologies (hypertension *vs* diabetes, hypertension *vs* chronic glomerulonephritis, and diabetes *vs* chronic glomerulonephritis), there was no statistical difference for KIM-1, NGAL, or NAG.

**Table 5:**
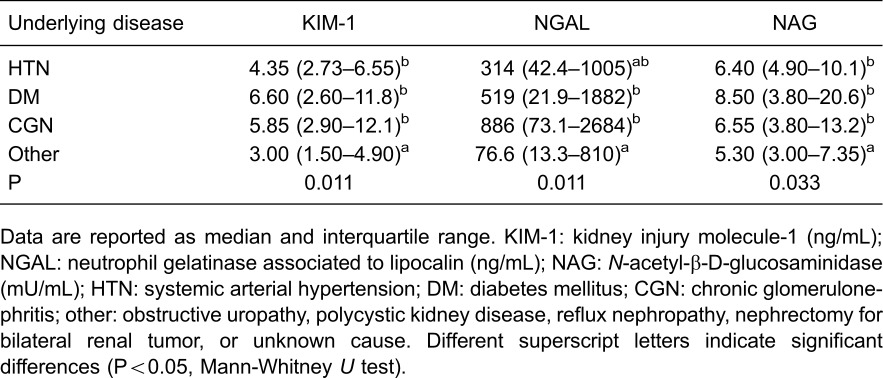
Association between underlying disease and urinary biomarkers in patients not on dialysis at baseline.

To control for factors independently associated with the adverse CKD progression outcomes, ESKD (dialysis or transplantation), or death in patients not on dialysis at baseline, univariate and multivariate Poisson regression analyses were performed (Table 6). After adjustment using the multivariate model, present or former tobacco smoking (P=0.044) and urinary NGAL (P=0.004) were the only predictors independently associated with poor outcomes. While smokers or former smokers had a 41.7% increase in risk of adverse outcomes, a 300-ng/mL increase in urinary NGAL levels resulted in an average increase of 2.2% in risk for these outcomes. KIM-1 and NAG were not associated with poor outcomes after a median follow-up of 15 months.

**Table 6:**
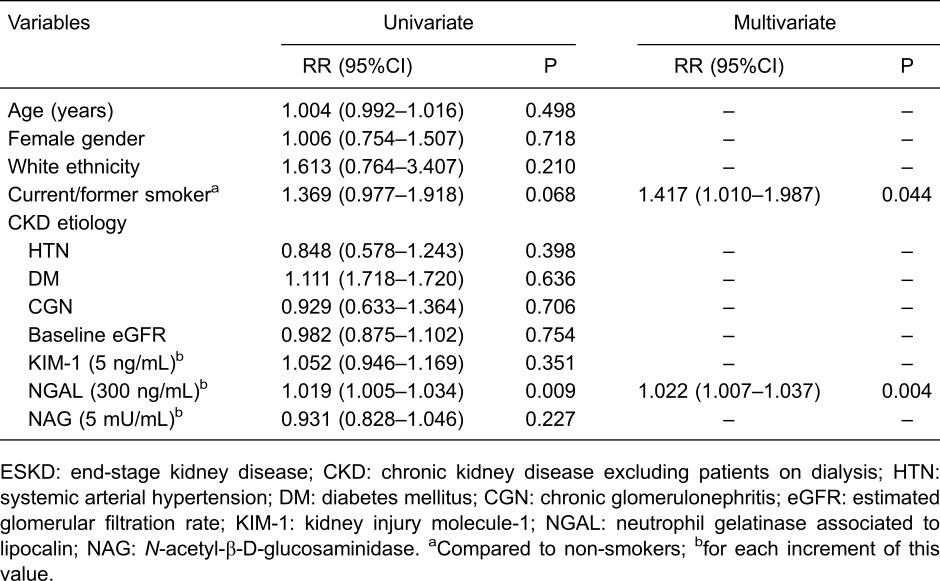
Poisson regression analysis to determine factors independently associated with adverse outcomes (progression of CKD stage, ESKD, or death) in patients not on dialysis at baseline.

Increasing age and urinary NGAL were associated with risk of death in patients who were on dialysis at study entry. For each 1-year increase in age, the risk of death increased by 4.7% (P=0.007), and each 300-ng/mL increase in urinary levels of NGAL resulted in an average increase of 1.3% in the risk of death (Table 7). KIM-1 and NAG were not associated with this outcome.

**Table 7:**
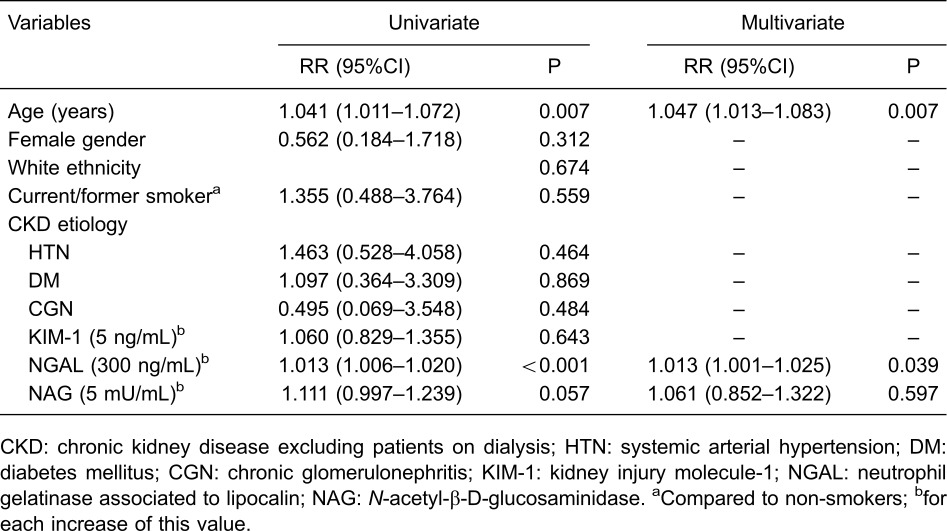
Poisson regression analysis to determine factors independently associated with death from any cause in patients on hemodialysis at baseline.

## Discussion

There is concern in the nephrology community about strategies for early identification of kidney damage in patients at risk of developing CKD. CKD affects more than 10% of the general population in the U.S. alone and is associated with high rates of all-cause and cardiovascular mortality (1), imposing a high cost to the public health services. Serum and urinary biomarkers that have been employed for decades to detect renal dysfunction and damage, such as creatinine and proteinuria respectively, cannot capture accurately the extension of the injury within the glomerular, tubular, or interstitial compartments, nor can they predict disease progression, renal survival, and mortality. In this scenario, novel biomarkers of CKD that could reflect early kidney damage and thus provide a window for early clinical intervention have been sought intensively.

Urinary KIM-1, NGAL, and NAG are expressed in response to ischemic, toxic, or inflammatory injuries to tubular epithelial cells and interstitium. This upregulation is predictive of kidney dysfunction and of adverse renal and cardiovascular outcomes. As examples, KIM-1 was associated with risk of rapid decline in kidney function and incident CKD stage 3 in the Multi-Ethnic Study of Atherosclerosis (21), independent of albuminuria levels. In the Chronic Renal Insufficiency Cohort Study (26), urinary levels of NGAL were independently associated with future ischemic atherosclerotic events in patients with CKD. High levels of NGAL in serum and urine predicted progression of kidney disease in several nephropathies, such as diabetic nephropathy (27), lupus nephritis (28), polycystic kidney disease (29), and IgA nephropathy (30). Plasma and urinary KIM-1 independently predicted renal function loss and risk of ESKD in a cohort of proteinuric patients with type 1 diabetes ([Bibr B23] 24), as well as in IgA nephropathy (30). Tissue and urinary KIM-1 levels were associated inversely with renal function and positively with inflammation and kidney tissue fibrosis (31), showing its potential to serve as a biomarker of active and progressive forms of various renal diseases and thus supplement routine clinical assessment in patients with CKD.

In the present study, patients in the highest tertile of KIM-1 had a higher proportion of progression to worse CKD stages, which was statistically significant. This association was not observed with the highest tertiles of NGAL or NAG. Moreover, the patients who progressed exhibited higher values of urinary KIM-1 and NGAL compared to non-progressors, but this difference was of borderline significance. The broad variability of NGAL values, as shown by the wide interquartile range, may have contributed to the lack of statistical significance. Bolignano et al. (22), studying a cohort of 96 patients with CKD from various etiologies, reported serum and urinary NGAL to be inversely correlated with eGFR; both markers predicted progression of renal impairment after a median follow-up of 18.5 months. We also found that, for each 300-ng/mL increase in urinary NGAL, there was an increased risk for the composite outcome CKD progression, ESKD, or death in CKD patients not on dialysis, and an increased risk of death in the dialysis patients. Despite the short observation period, our findings are in line with those of the studies mentioned above, suggesting that KIM-1 and NGAL can predict CKD progression and adverse renal outcomes.

Recently, Zhou et al. (32) published a meta-analysis involving 10 prospective studies with 29,366 participants, reporting the clinical utility of urinary concentration of NGAL as an independent predictor of ESKD among patients with CKD (pooled adjusted RR: 1.40; 95%CI=121–1.61; P<0.001); urinary KIM-1 exhibited a borderline significance (pooled adjusted RR: 1.13; 95%CI=1.00–1.27; P=0.057) as an independent predictive factor of CKD stage 3. No significant statistical heterogeneity was identified among the studies for both analyses. These data reinforce our findings, emphasizing the potential clinical use of these two biomarkers to predict poor renal outcomes, and perhaps to identify at-risk patients at an early disease stage.

How can increased urinary NGAL concentrations reflect early structural changes in renal compartments, as troponin does in acute myocardial injury, and be predictive of renal outcomes? The NGAL molecule is freely filtered by the glomerulus and reabsorbed in the proximal tubules; its secretion in urine occurs after direct renal insults. According to Bolignano et al. (17), in chronically damaged tubular cells, high quantities of NGAL are produced due to intracellular stress and protein overload, perhaps as a compensatory response to counteract intracellular complement-induced oxidative stress. At the molecular level, Kuwabara et al. (33) showed, in mouse experimental models of diabetes, glomerulonephritis, and interstitial nephritis, that elevated NGAL appeared to derive from impaired reabsorption in proximal tubules, whereas in obstructed kidneys the site of accumulation was the distal nephron. These authors suggested that both glomerular filtration and local synthesis are mechanisms by which NGAL reflects damage to glomeruli, proximal tubules, and distal nephrons. In our cohort, the lowest and highest levels of NGAL, KIM-1, and NAG were found in stage 1 and stage 5d patients respectively; this is consistent with expectations regarding the severity and extent of structural kidney damage in less or more advanced CKD.

KIM-1 is one of the most widely studied biomarkers of proximal tubular injury, both in AKI and in CKD (15,16,20,24,25,30,31). As reviewed by Bonventre and Yang (34), KIM-1 mediates epithelial phagocytosis after injury, converting the proximal tubular cell into a phagocyte and thus modulating the immune response and repair of damaged cells. Blood and urinary levels of this molecule correlate with albuminuria (25) and severity of kidney injury, and predict the development of AKI in various settings (34). In type 1 diabetes, lower baseline levels of urinary KIM-1 and NAG are associated with regression of microalbuminuria (35). Interestingly, the combination of urinary KIM-1, NGAL, and NAG values was found to predict AKI after cardiac surgery, with enhanced sensitivity to early detect postoperative acute renal injury compared with each biomarker alone (36). Experimentally, KIM-1 gene expression reflects ongoing damage in different segments of the renal cortex and in the tubulointerstitium (37). In the clinical setting, blood and urinary KIM-1 levels have been found to predict renal function decline, progression of CKD, and development of ESKD (21,25). In the present study, KIM-1 correlated with CKD stages, and a greater proportion of patients in the highest tertile of KIM-1 exhibited progression of CKD (57.8 vs 37% in the lowest tertile, P=0.047), providing further evidence of the utility of KIM-1 as a biomarker of progressive chronic kidney damage. As another example, in renal grafts, KIM-1 mRNA signaling was increased in biopsies with interstitial fibrosis and tubular atrophy (IF/TA) *vs* in specimens with acute calcineurin inhibitor toxicity or acute rejection, and KIM-1 mRNA in urine cells was also increased in patients with IF/TA, which shows its potential as a biomarker of injuries that can trigger intragraft fibrosis (38).

NAG, the oldest-known of our three biomarkers of interest, is a lysosomal enzyme found in the microvilli of proximal tubule epithelial cells. In patients with tubular and interstitial renal impairment, total urinary NAG activity is elevated, as demonstrated in various renal diseases, including proteinuric glomerulopathies (39) and diabetes (25). In American Indians with type 2 diabetes, urinary NAG was found to correlate with albuminuria levels, but not with incident ESKD or mortality (25). In the Diabetes Control and Complications Trial (40), baseline urinary NAG independently predicted macroalbuminuria and microalbuminuria, identifying subjects with type 1 diabetes at risk for the development of nephropathy. We could not demonstrate an association between urinary NAG and progression of CKD or development of adverse renal outcomes. Overall, it seems that NAG does not perform as well as KIM-1 or NGAL as a marker of progressive chronic damage or as a predictive factor for worse outcomes, but behaves similarly to these other biomarkers as an indicator of renal injury, such as AKI after cardiac surgery (36).

No reliable association could be found between the tested urinary biomarkers and type of renal disease. Urinary concentrations of these biomarkers did not differ across the most common etiologies of CKD (hypertension, diabetes, or chronic glomerulonephritis). In other etiologies, which were predominantly tubulointerstitial (e.g., obstructive uropathy, polycystic kidney disease, nephrolithiasis) but were underrepresented in our cohort, levels of these biomarkers were significantly lower. This warrants further investigation in a larger sample.

An important limitation of this study is the short follow-up time. Even though we could detect a significant association of increasing NGAL and KIM-1 levels with CKD progression, a longer follow-up would be needed to demonstrate more robust correlations between these urinary biomarkers and poor renal and cardiovascular outcomes. Of equal importance, reference values of urinary biomarkers above which kidney damage must be ongoing need to be established for routine use in clinical practice, particularly in the early stages of CKD, when therapeutic interventions are most effective. Another relevant limitation of the study is related to the number of CKD patients not on dialysis (n=139), which is inferior to the calculated sample size; this increases the chance of type II error regarding the outcome ESKD, although not in relation to the combined outcome ESKD or death. Further, a potential limitation is the prolonged sample storage time until biomarker measurement. However, as demonstrated previously (25,36), the stability of KIM-1 and NGAL was not significantly affected in urine samples stored for 2 years at –80°C with no more than two freeze-thaw cycles.

In conclusion, this study detected a trend toward increased baseline levels of KIM-1 and NGAL in patients with renal impairment who progressed to more advanced stages of CKD. Urinary NGAL was predictive of CKD progression, ESKD, and death in non-dialyzed patients, and was associated with increased risk of death in patients on dialysis patients. The clinical implications of these findings should be explored further in a longer follow-up.
